# Kinetic Theory-Based Methods in Fluid Dynamics

**DOI:** 10.3390/e25020255

**Published:** 2023-01-31

**Authors:** Zhen Chen, Liangqi Zhang, Liming Yang

**Affiliations:** 1 School of Naval Architecture, Ocean and Civil Engineering, Shanghai Jiao Tong University, Shanghai 200240, China; 2 College of Aerospace Engineering, Chongqing University, Chongqing 400044, China; 3 Department of Aerodynamics, Nanjing University of Aeronautics and Astronautics, Nanjing 210016, China

Kinetic theory stems from the statistical mechanics established at the mesoscopic scale. In the area of fluid dynamics, kinetic theory outperforms macroscopic interpretations (represented by the Navier–Stokes equations) in multiple aspects: it provides theoretical generality with no restrictions from the continuum assumption, clear interpretation of the streaming and collision of fluid particles in a physical process, simple algebraic formulas instead of partial differential equations in numerical evolution, and convenient implementation in parallel computation. Various methods, such as the discrete velocity method, gas kinetic scheme, unified gas kinetic scheme, lattice Boltzmann method, etc., have been developed within the framework of kinetic theory. These methods play unique and important roles in almost all studies of fluid dynamics. However, their broader application to engineering problems is often hindered by intrinsic limitations. Kinetic theory-based methods usually consume larger virtual memory than macroscopic methods. Additionally, high-fidelity simulations of flows beyond the continuum regime are still time-consuming. Therefore, developing robust and efficient kinetic theory-based methods is an urgent need in the fluid dynamics community.

This Special Issue is a timely forum for presenting recent advances in the very active area of kinetic theory-based methods in fluid dynamics. After a year-long preparation and a rigorous peer-review process, 12 articles were finally accepted for publication in this Special Issue. These articles report the latest developments in kinetic-theory-related numerical schemes [1,2] and typical applications in multiphase flows [3], thermal flows [4], micro/nano flows [5,6], flows in porous media [7], and compressible flows [8,9], as well as other areas of fluid dynamics [10,11,12]. Specifically, Song et al. [1] proposed a simplified linearized Boltzmann method for the effective simulation of acoustic propagation with a lower cost of virtual memory. Xiao [2] developed a well-balanced unified gas-kinetic scheme to model the dynamics of multicomponent gaseous flows under gravity, which allows for evolving a gravitational system under any initial condition to the hydrostatic equilibrium, and thus could be a proper solver for long-term evolving systems such as galaxy formation. Yang et al. [3] managed to remove the force imbalance in the direct implementation of a lattice Boltzmann free-energy model on the discrete unified gas kinetic scheme and successfully derived a robust free-energy model for van der Waals fluid. Feng et al. [4] utilized the multiple-relaxation-time lattice Boltzmann method to investigate the thermal behaviors of convection melting in metal foam under sinusoidal temperature boundary conditions. Wu and Zhou [5] presented an application of the lattice Boltzmann flux solver to the modelling of the natural convection process within a square cavity filled by nanofluid. Guo and Hou [6] derived an anisotropic slip boundary condition based on nonlinear velocity profiles near the wall, consolidated this new boundary treatment into the discrete unified gas kinetic scheme, and investigated the effects of anisotropic slip on the two-sided orthogonal oscillating micro-lid-driven cavity flow through three-dimensional simulations. Liu et al. [7] carried out a series of simulations using the smoothed particle hydrodynamics method to shed light on the physics under the imbibition phenomenon in porous media. Zhou et al. [8] employed the gas-kinetic BGK scheme and performed a thorough analysis of the thermal protection system for vehicles operating in extreme conditions of hypersonic flows. Jiang et al. [9] investigated the aerodynamic characteristics of an X38-like vehicle considering strong viscous interactions and complicated rarified effects, which could be of reference value to engineering designs. Morozov and Titarev [10] utilized three numerical tools to study the dynamics of gas expansion due to intense nanosecond laser evaporation into vacuum, with specific attention paid to factors that are essential for experimental measurements. Megías and Santos [11] established a numerical model to interpret interactions between the dilute granular gases and a thermal bath made from smaller particles, and found that the Sonine approximation performs better than the Maxwellian approximation in revealing inelasticity, drag nonlinearity and memory effects. Qi et al. [12] employed an immersed boundary-lattice Boltzmann method to simulate self-propelled particles in a simple shear flow, and studied the effects of multiple flow parameters (swimming Reynolds number, flow Reynolds number and blocking rate) on the kinematics and flow patterns. 

The Guest Editors would like to express their sincere gratitude to all authors for their valuable contributions which made this Special Issue possible, and to all anonymous referees for their valuable time and professional feedback which substantially improved the quality of this Special Issue. Special thanks are given to the editorial team of *Entropy* for their consistent support and valuable assistance.
